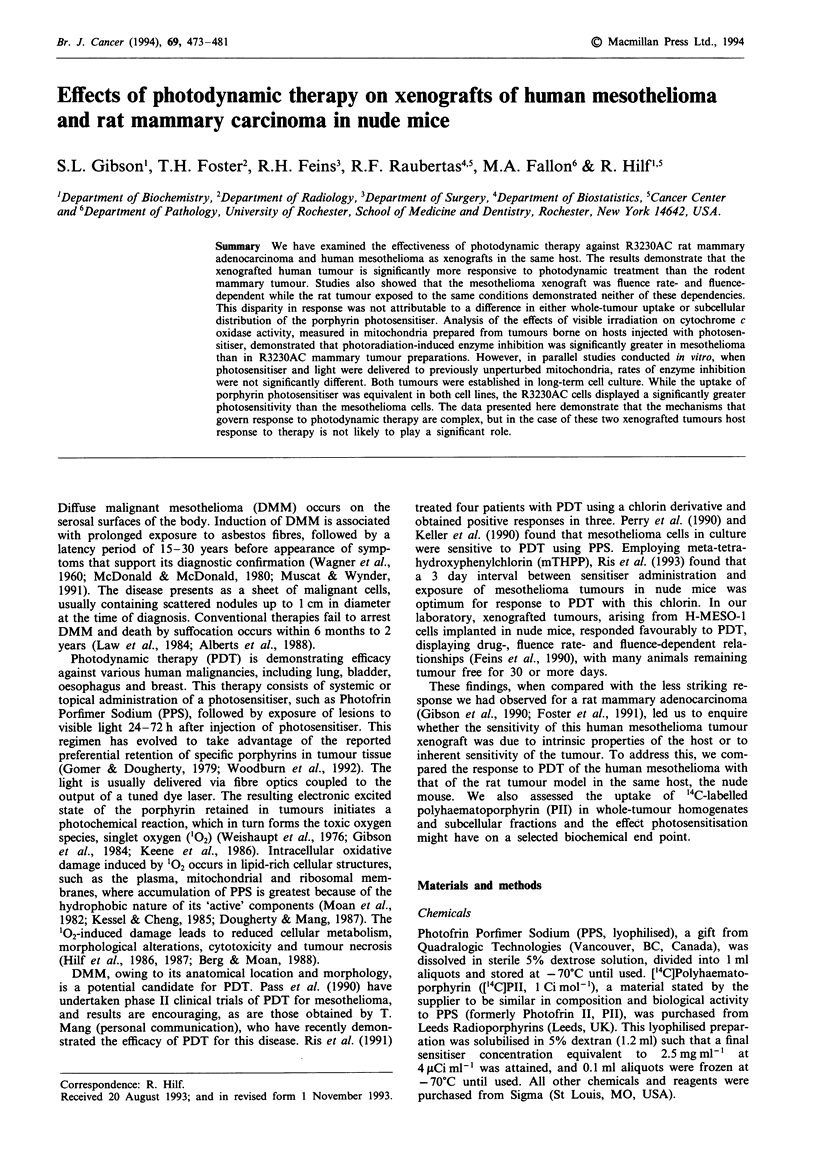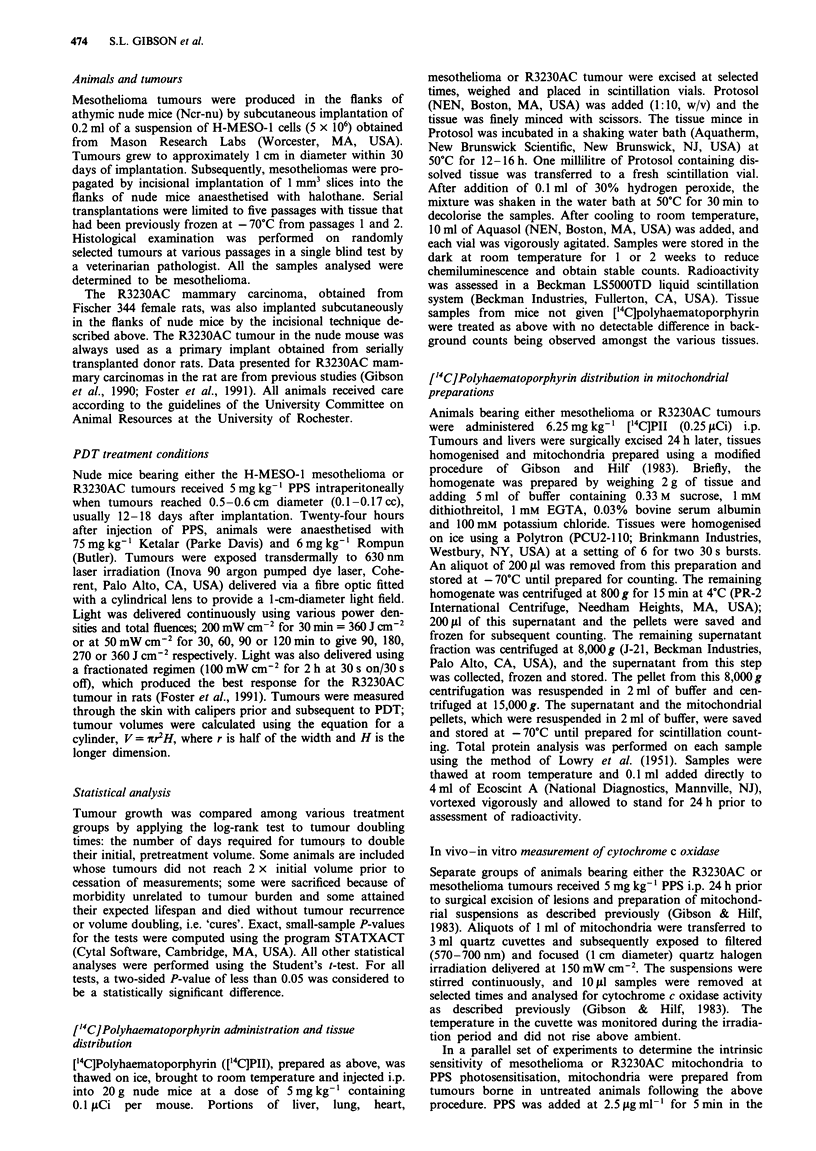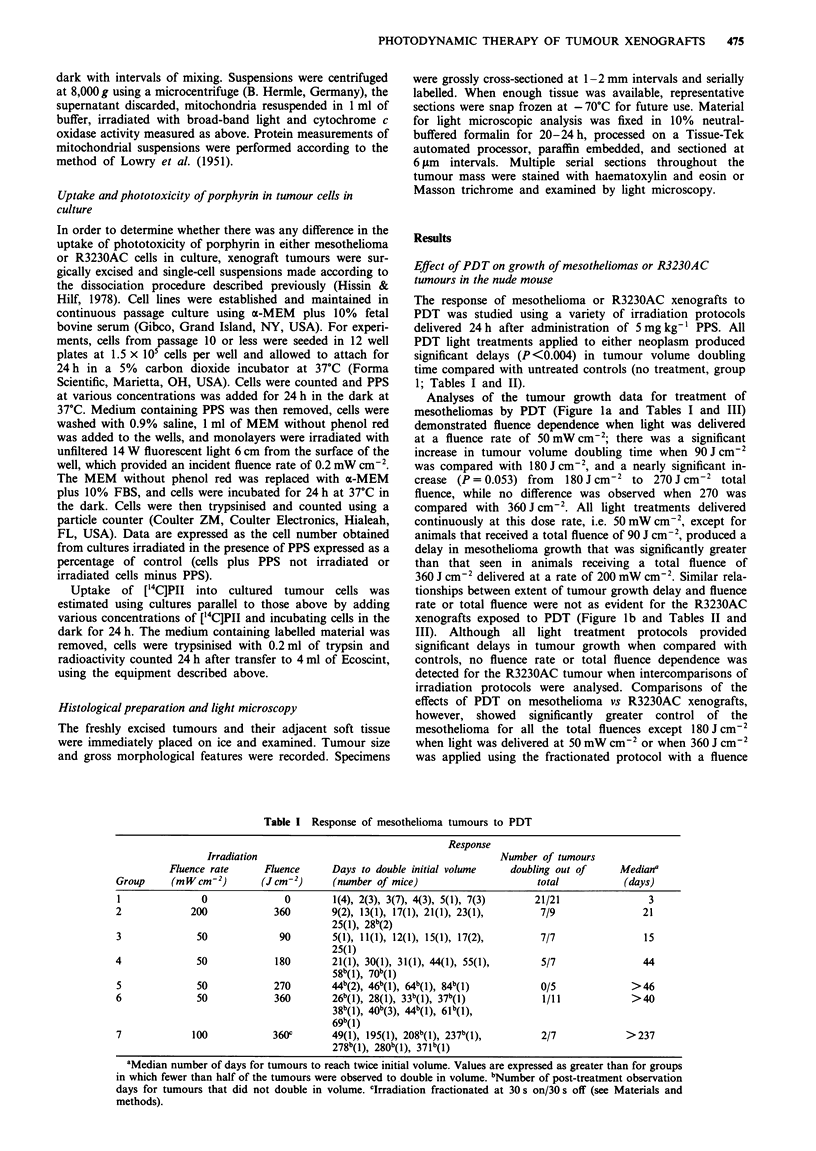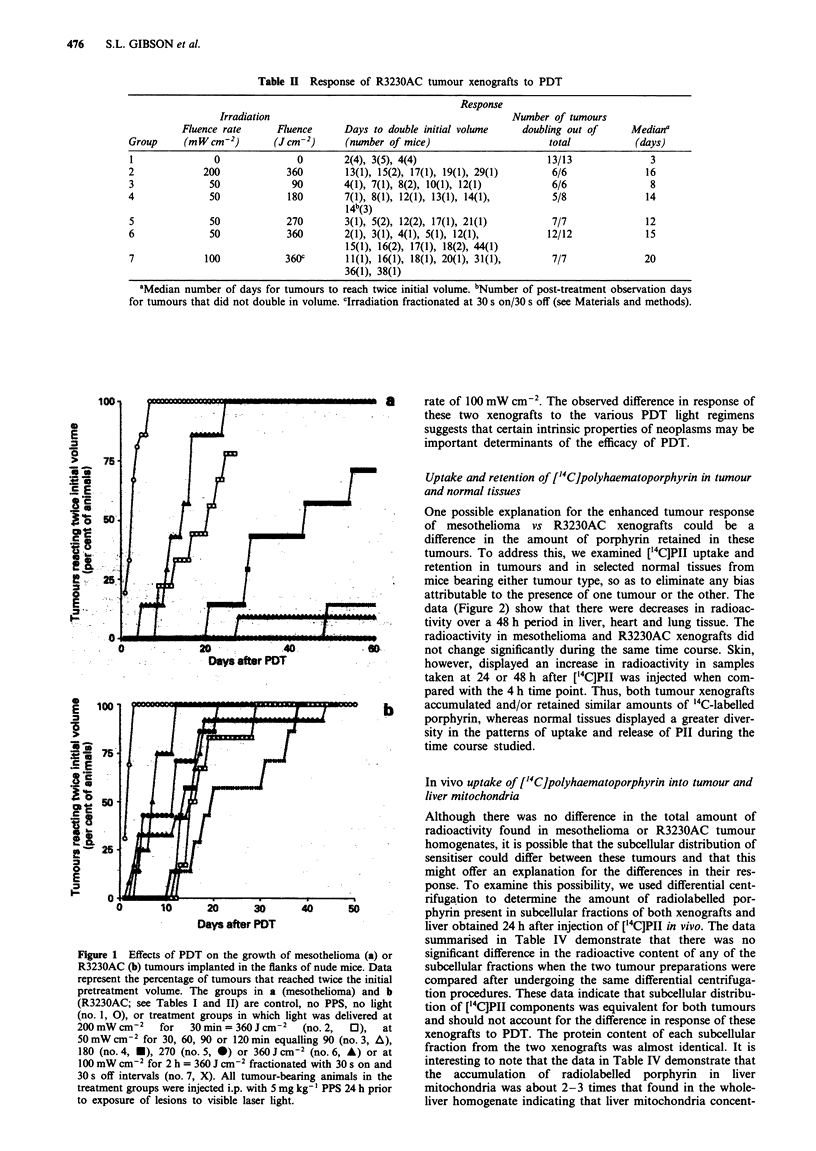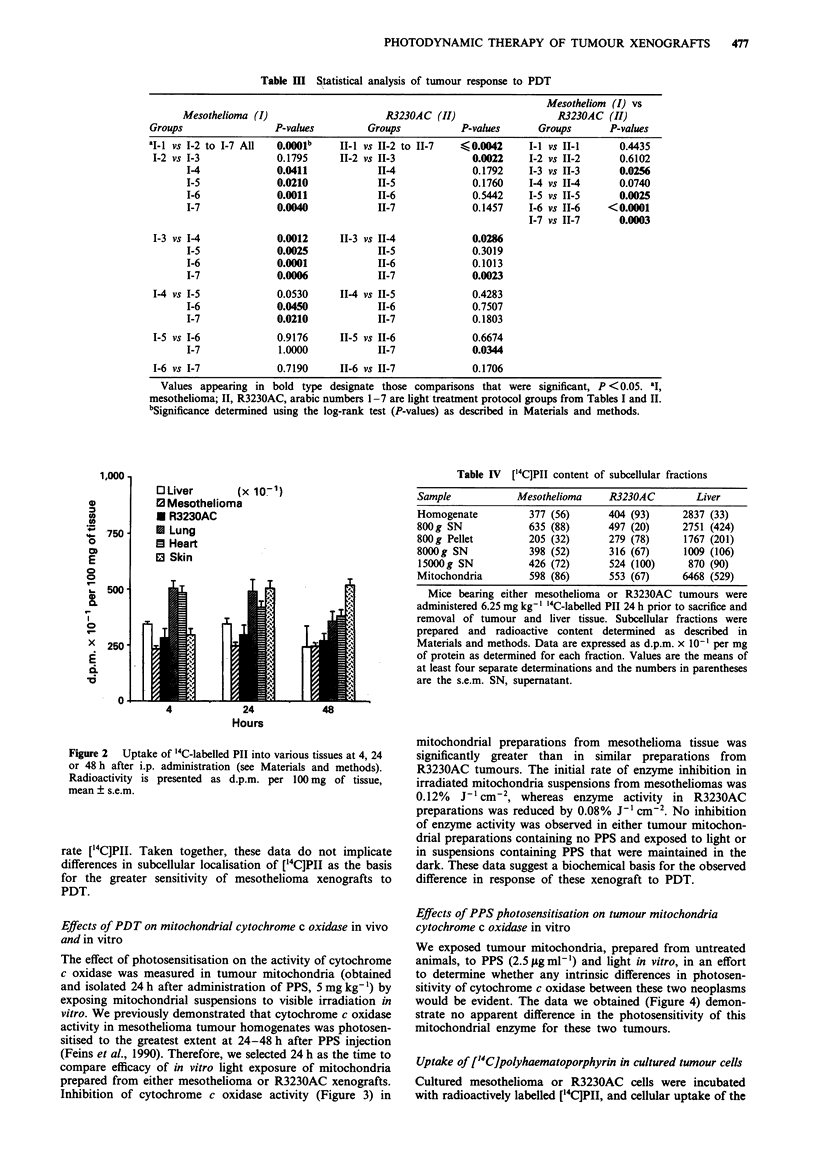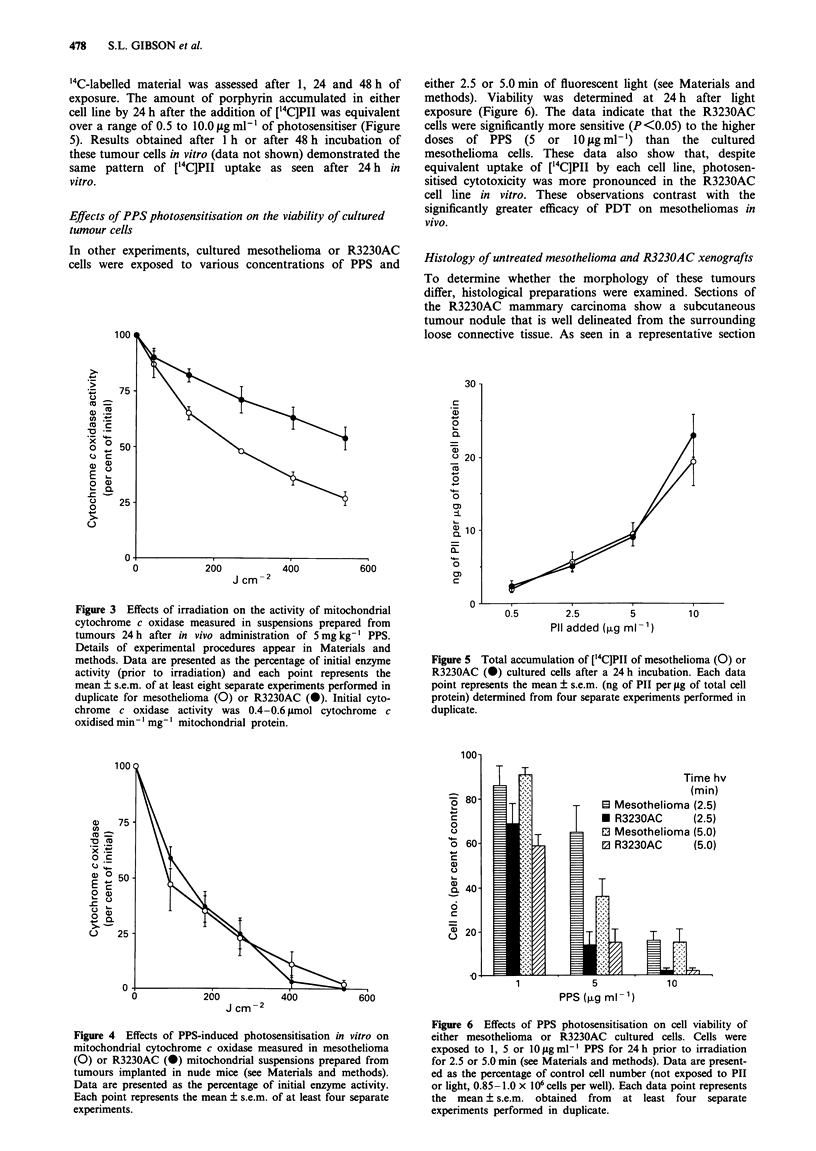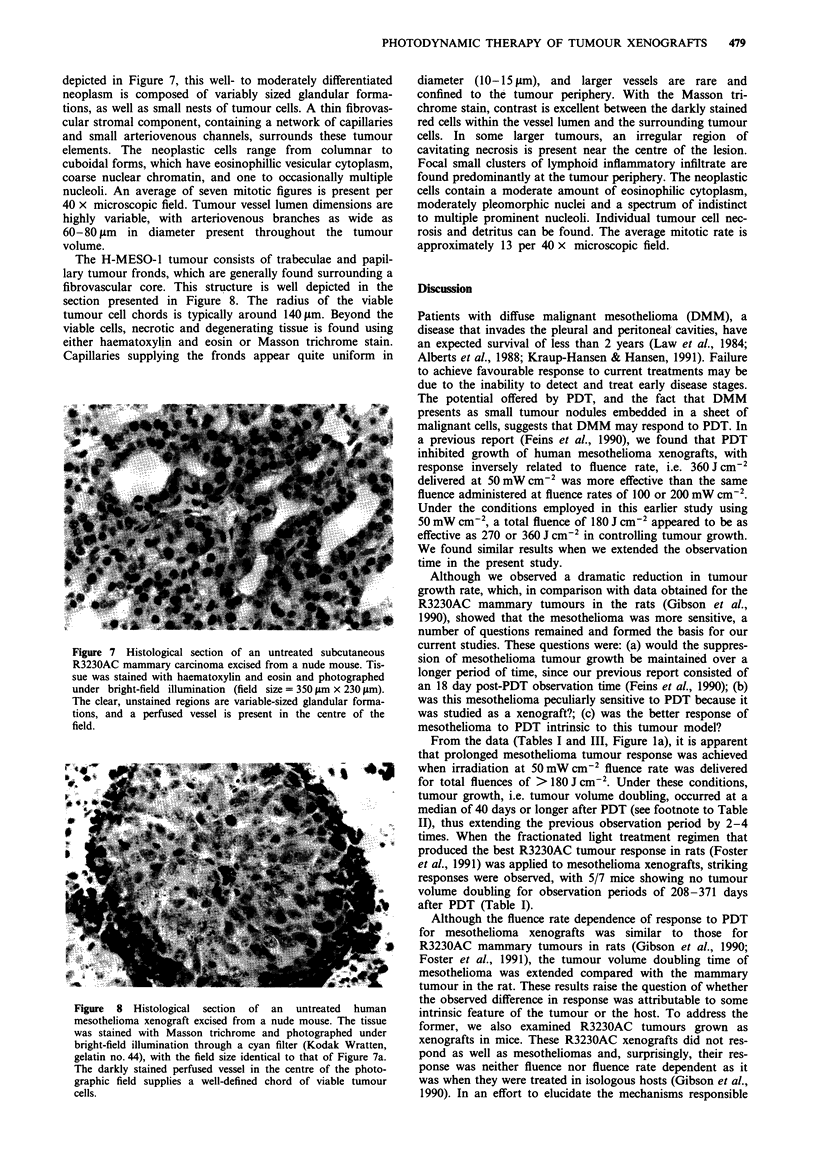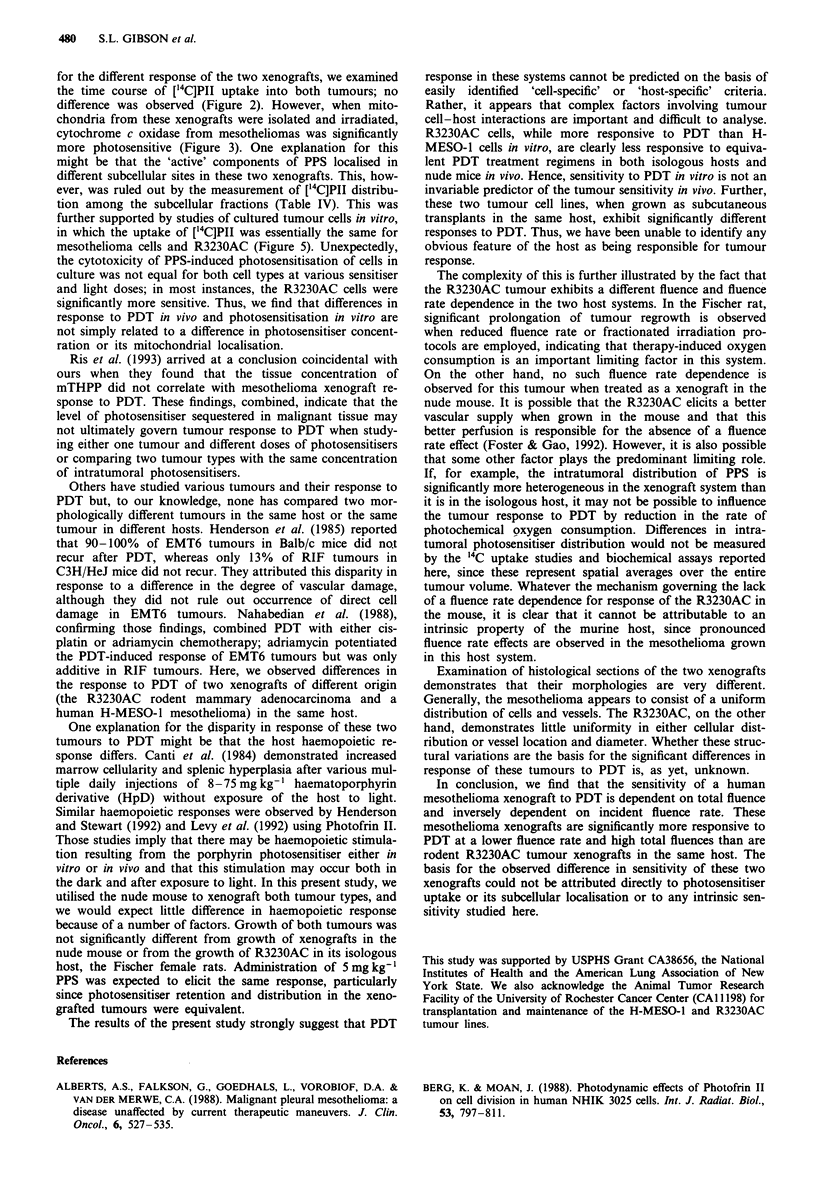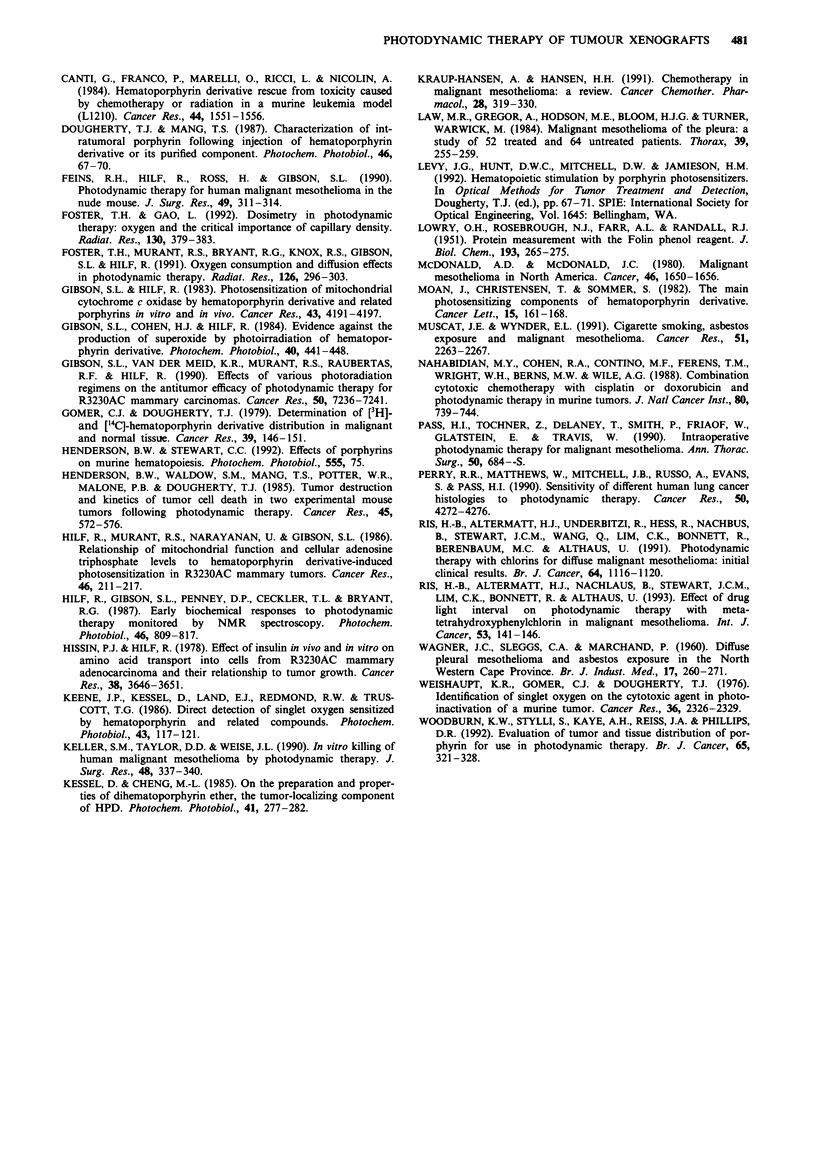# Effects of photodynamic therapy on xenografts of human mesothelioma and rat mammary carcinoma in nude mice.

**DOI:** 10.1038/bjc.1994.86

**Published:** 1994-03

**Authors:** S. L. Gibson, T. H. Foster, R. H. Feins, R. F. Raubertas, M. A. Fallon, R. Hilf

**Affiliations:** Department of Biochemistry, University of Rochester, School of Medicine and Dentistry, New York 14642.

## Abstract

**Images:**


Br. J. Cancer (1994), 69, 473–481 © Macmillan Press Ltd., 1994

### Effects of photodynamic therapy on xenografts of human mesothelioma and rat mammary carcinoma in nude mice

S.L. Gibson[1], T.H. Foster[2], R.H. Feins[3], R.F. Raubertas[4,5], M.A. Fallon[6] & R. Hilf[1,5]

[1]*Department of Biochemistry,* [2]*Department of Radiology,* [3]*Department of Surgery,* [4]*Department of Biostatistics,* [5]*Cancer Center and* [6]*Department of Pathology, University of Rochester, School of Medicine and Dentistry, Rochester, New York 14642, USA.*

**Summary** We have examined the effectiveness of photodynamic therapy against R3230AC rat mammary adenocarcinoma and human mesothelioma as xenografts in the same host. The results demonstrate that the xenografted human tumour is significantly more responsive to photodynamic treatment than the rodent mammary tumour. Studies also showed that the mesothelioma xenograft was fluence rate- and fluence-dependent while the rat tumour exposed to the same conditions demonstrated neither of these dependencies. This disparity in response was not attributable to a difference in either whole-tumour uptake or subcellular distribution of the porphyrin photosensitiser. Analysis of the effects of visible irradiation on cytochrome *c* oxidase activity, measured in mitochondria prepared from tumours borne on hosts injected with photosensitiser, demonstrated that photoradiation-induced enzyme inhibition was significantly greater in mesothelioma than in R3230AC mammary tumour preparations. However, in parallel studies conducted *in vitro*, when photosensitiser and light were delivered to previously unperturbed mitochondria, rates of enzyme inhibition were not significantly different. Both tumours were established in long-term cell culture. While the uptake of porphyrin photosensitiser was equivalent in both cell lines, the R3230AC cells displayed a significantly greater photosensitivity than the mesothelioma cells. The data presented here demonstrate that the mechanisms that govern response to photodynamic therapy are complex, but in the case of these two xenografted tumours host response to therapy is not likely to play a significant role.

Diffuse malignant mesothelioma (DMM) occurs on the serosal surfaces of the body. Induction of DMM is associated with prolonged exposure to asbestos fibres, followed by a latency period of 15–30 years before appearance of symptoms that support its diagnostic confirmation (Wagner *et al.*, 1960; McDonald & McDonald, 1980; Muscat & Wynder, 1991). The disease presents as a sheet of malignant cells, usually containing scattered nodules up to 1 cm in diameter at the time of diagnosis. Conventional therapies fail to arrest DMM and death by suffocation occurs within 6 months to 2 years (Law *et al.*, 1984; Alberts *et al.*, 1988).

Photodynamic therapy (PDT) is demonstrating efficacy against various human malignancies, including lung, bladder, oesophagus and breast. This therapy consists of systemic or topical administration of a photosensitiser, such as Photofrin Porfimer Sodium (PPS), followed by exposure of lesions to visible light 24–72 h after injection of photosensitiser. This regimen has evolved to take advantage of the reported preferential retention of specific porphyrins in tumour tissue (Gomer & Dougherty, 1979; Woodburn *et al.*, 1992). The light is usually delivered via fibre optics coupled to the output of a tuned dye laser. The resulting electronic excited state of the porphyrin retained in tumours initiates a photochemical reaction, which in turn forms the toxic oxygen species, singlet oxygen ($^1O_2$) (Weishaupt *et al.*, 1976; Gibson *et al.*, 1984; Keene *et al.*, 1986). Intracellular oxidative damage induced by $^1O_2$ occurs in lipid-rich cellular structures, such as the plasma, mitochondrial and ribosomal membranes, where accumulation of PPS is greatest because of the hydrophobic nature of its 'active' components (Moan *et al.*, 1982; Kessel & Cheng, 1985; Dougherty & Mang, 1987). The $^1O_2$-induced damage leads to reduced cellular metabolism, morphological alterations, cytotoxicity and tumour necrosis (Hilf *et al.*, 1986, 1987; Berg & Moan, 1988).

DMM, owing to its anatomical location and morphology, is a potential candidate for PDT. Pass *et al.* (1990) have undertaken phase II clinical trials of PDT for mesothelioma, and results are encouraging, as are those obtained by T. Mang (personal communication), who have recently demonstrated the efficacy of PDT for this disease. Ris *et al.* (1991) treated four patients with PDT using a chlorin derivative and obtained positive responses in three. Perry *et al.* (1990) and Keller *et al.* (1990) found that mesothelioma cells in culture were sensitive to PDT using PPS. Employing meta-tetrahydroxyphenylchlorin (mTHPP), Ris *et al.* (1993) found that a 3 day interval between sensitiser administration and exposure of mesothelioma tumours in nude mice was optimum for response to PDT with this chlorin. In our laboratory, xenografted tumours, arising from H-MESO-1 cells implanted in nude mice, responded favourably to PDT, displaying drug-, fluence rate- and fluence-dependent relationships (Feins *et al.*, 1990), with many animals remaining tumour free for 30 or more days.

These findings, when compared with the less striking response we had observed for a rat mammary adenocarcinoma (Gibson *et al.*, 1990; Foster *et al.*, 1991), led us to enquire whether the sensitivity of this human mesothelioma tumour xenograft was due to intrinsic properties of the host or to inherent sensitivity of the tumour. To address this, we compared the response to PDT of the human mesothelioma with that of the rat tumour model in the same host, the nude mouse. We also assessed the uptake of $^{14}C$-labelled polyhaematoporphyrin (PII) in whole-tumour homogenates and subcellular fractions and the effect photosensitisation might have on a selected biochemical end point.

### Materials and methods

### Chemicals

Photofrin Porfimer Sodium (PPS, lyophilised), a gift from Quadralogic Technologies (Vancouver, BC, Canada), was dissolved in sterile 5% dextrose solution, divided into 1 ml aliquots and stored at $-70°C$ until used. [$^{14}C$]Polyhaematoporphyrin ([$^{14}C$]PII, 1 Ci mol$^{-1}$), a material stated by the supplier to be similar in composition and biological activity to PPS (formerly Photofrin II, PII), was purchased from Leeds Radioporphyrins (Leeds, UK). This lyophilised preparation was solubilised in 5% dextran (1.2 ml) such that a final sensitiser concentration equivalent to 2.5 mg ml$^{-1}$ at 4 $\mu$Ci ml$^{-1}$ was attained, and 0.1 ml aliquots were frozen at $-70°C$ until used. All other chemicals and reagents were purchased from Sigma (St Louis, MO, USA).

Correspondence: R. Hilf.
Received 20 August 1993; and in revised form 1 November 1993.

*Animals and tumours*

Mesothelioma tumours were produced in the flanks of athymic nude mice (Ncr-nu) by subcutaneous implantation of 0.2 ml of a suspension of H-MESO-1 cells ($5 \times 10^6$) obtained from Mason Research Labs (Worcester, MA, USA). Tumours grew to approximately 1 cm in diameter within 30 days of implantation. Subsequently, mesotheliomas were propagated by incisional implantation of 1 $mm^3$ slices into the flanks of nude mice anaesthetised with halothane. Serial transplantations were limited to five passages with tissue that had been previously frozen at $-70$°C from passages 1 and 2. Histological examination was performed on randomly selected tumours at various passages in a single blind test by a veterinarian pathologist. All the samples analysed were determined to be mesothelioma.

The R3230AC mammary carcinoma, obtained from Fischer 344 female rats, was also implanted subcutaneously in the flanks of nude mice by the incisional technique described above. The R3230AC tumour in the nude mouse was always used as a primary implant obtained from serially transplanted donor rats. Data presented for R3230AC mammary carcinomas in the rat are from previous studies (Gibson *et al.*, 1990; Foster *et al.*, 1991). All animals received care according to the guidelines of the University Committee on Animal Resources at the University of Rochester.

*PDT treatment conditions*

Nude mice bearing either the H-MESO-1 mesothelioma or R3230AC tumours received 5 mg $kg^{-1}$ PPS intraperitoneally when tumours reached 0.5–0.6 cm diameter (0.1–0.17 cc), usually 12–18 days after implantation. Twenty-four hours after injection of PPS, animals were anaesthetised with 75 mg $kg^{-1}$ Ketalar (Parke Davis) and 6 mg $kg^{-1}$ Rompun (Butler). Tumours were exposed transdermally to 630 nm laser irradiation (Inova 90 argon pumped dye laser, Coherent, Palo Alto, CA, USA) delivered via a fibre optic fitted with a cylindrical lens to provide a 1-cm-diameter light field. Light was delivered continuously using various power densities and total fluences; 200 mW $cm^{-2}$ for 30 min = 360 J $cm^{-2}$ or at 50 mW $cm^{-2}$ for 30, 60, 90 or 120 min to give 90, 180, 270 or 360 J $cm^{-2}$ respectively. Light was also delivered using a fractionated regimen (100 mW $cm^{-2}$ for 2 h at 30 s on/30 s off), which produced the best response for the R3230AC tumour in rats (Foster *et al.*, 1991). Tumours were measured through the skin with calipers prior and subsequent to PDT; tumour volumes were calculated using the equation for a cylinder, $V = \pi r^2 H$, where $r$ is half of the width and $H$ is the longer dimension.

*Statistical analysis*

Tumour growth was compared among various treatment groups by applying the log-rank test to tumour doubling times: the number of days required for tumours to double their initial, pretreatment volume. Some animals are included whose tumours did not reach 2 × initial volume prior to cessation of measurements; some were sacrificed because of morbidity unrelated to tumour burden and some attained their expected lifespan and died without tumour recurrence or volume doubling, i.e. 'cures'. Exact, small-sample *P*-values for the tests were computed using the program STATXACT (Cytal Software, Cambridge, MA, USA). All other statistical analyses were performed using the Student's *t*-test. For all tests, a two-sided *P*-value of less than 0.05 was considered to be a statistically significant difference.

*[$^{14}$C]Polyhaematoporphyrin administration and tissue distribution*

[$^{14}$C]Polyhaematoporphyrin ([$^{14}$C]PII), prepared as above, was thawed on ice, brought to room temperature and injected i.p. into 20 g nude mice at a dose of 5 mg $kg^{-1}$ containing 0.1 $\mu$Ci per mouse. Portions of liver, lung, heart, mesothelioma or R3230AC tumour were excised at selected times, weighed and placed in scintillation vials. Protosol (NEN, Boston, MA, USA) was added (1:10, w/v) and the tissue was finely minced with scissors. The tissue mince in Protosol was incubated in a shaking water bath (Aquatherm, New Brunswick Scientific, New Brunswick, NJ, USA) at 50°C for 12–16 h. One millilitre of Protosol containing dissolved tissue was transferred to a fresh scintillation vial. After addition of 0.1 ml of 30% hydrogen peroxide, the mixture was shaken in the water bath at 50°C for 30 min to decolorise the samples. After cooling to room temperature, 10 ml of Aquasol (NEN, Boston, MA, USA) was added, and each vial was vigorously agitated. Samples were stored in the dark at room temperature for 1 or 2 weeks to reduce chemiluminescence and obtain stable counts. Radioactivity was assessed in a Beckman LS5000TD liquid scintillation system (Beckman Industries, Fullerton, CA, USA). Tissue samples from mice not given [$^{14}$C]polyhaematoporphyrin were treated as above with no detectable difference in background counts being observed amongst the various tissues.

*[$^{14}$C]Polyhaematoporphyrin distribution in mitochondrial preparations*

Animals bearing either mesothelioma or R3230AC tumours were administered 6.25 mg $kg^{-1}$ [$^{14}$C]PII (0.25 $\mu$Ci) i.p. Tumours and livers were surgically excised 24 h later, tissues homogenised and mitochondria prepared using a modified procedure of Gibson and Hilf (1983). Briefly, the homogenate was prepared by weighing 2 g of tissue and adding 5 ml of buffer containing 0.33 M sucrose, 1 mM dithiothreitol, 1 mM EGTA, 0.03% bovine serum albumin and 100 mM potassium chloride. Tissues were homogenised on ice using a Polytron (PCU2-110; Brinkmann Industries, Westbury, NY, USA) at a setting of 6 for two 30 s bursts. An aliquot of 200 $\mu$l was removed from this preparation and stored at $-70$°C until prepared for counting. The remaining homogenate was centrifuged at 800 ***g*** for 15 min at 4°C (PR-2 International Centrifuge, Needham Heights, MA, USA); 200 $\mu$l of this supernatant and the pellets were saved and frozen for subsequent counting. The remaining supernatant fraction was centrifuged at 8,000 ***g*** (J-21, Beckman Industries, Palo Alto, CA, USA), and the supernatant from this step was collected, frozen and stored. The pellet from this 8,000 ***g*** centrifugation was resuspended in 2 ml of buffer and centrifuged at 15,000 ***g***. The supernatant and the mitochondrial pellets, which were resuspended in 2 ml of buffer, were saved and stored at $-70$°C until prepared for scintillation counting. Total protein analysis was performed on each sample using the method of Lowry *et al.* (1951). Samples were thawed at room temperature and 0.1 ml added directly to 4 ml of Ecoscint A (National Diagnostics, Mannville, NJ), vortexed vigorously and allowed to stand for 24 h prior to assessment of radioactivity.

In vivo–in vitro *measurement of cytochrome* c *oxidase*

Separate groups of animals bearing either the R3230AC or mesothelioma tumours received 5 mg $kg^{-1}$ PPS i.p. 24 h prior to surgical excision of lesions and preparation of mitochondrial suspensions as described previously (Gibson & Hilf, 1983). Aliquots of 1 ml of mitochondria were transferred to 3 ml quartz cuvettes and subsequently exposed to filtered (570–700 nm) and focused (1 cm diameter) quartz halogen irradiation delivered at 150 mW $cm^{-2}$. The suspensions were stirred continuously, and 10 $\mu$l samples were removed at selected times and analysed for cytochrome *c* oxidase activity as described previously (Gibson & Hilf, 1983). The temperature in the cuvette was monitored during the irradiation period and did not rise above ambient.

In a parallel set of experiments to determine the intrinsic sensitivity of mesothelioma or R3230AC mitochondria to PPS photosensitisation, mitochondria were prepared from tumours borne in untreated animals following the above procedure. PPS was added at 2.5 $\mu$g $ml^{-1}$ for 5 min in the dark with intervals of mixing. Suspensions were centrifuged at 8,000 *g* using a microcentrifuge (B. Hermle, Germany), the supernatant discarded, mitochondria resuspended in 1 ml of buffer, irradiated with broad-band light and cytochrome *c* oxidase activity measured as above. Protein measurements of mitochondrial suspensions were performed according to the method of Lowry *et al.* (1951).

### *Uptake and phototoxicity of porphyrin in tumour cells in culture*

In order to determine whether there was any difference in the uptake of phototoxicity of porphyrin in either mesothelioma or R3230AC cells in culture, xenograft tumours were surgically excised and single-cell suspensions made according to the dissociation procedure described previously (Hissin & Hilf, 1978). Cell lines were established and maintained in continuous passage culture using α-MEM plus 10% fetal bovine serum (Gibco, Grand Island, NY, USA). For experiments, cells from passage 10 or less were seeded in 12 well plates at $1.5 \times 10^5$ cells per well and allowed to attach for 24 h in a 5% carbon dioxide incubator at 37°C (Forma Scientific, Marietta, OH, USA). Cells were counted and PPS at various concentrations was added for 24 h in the dark at 37°C. Medium containing PPS was then removed, cells were washed with 0.9% saline, 1 ml of MEM without phenol red was added to the wells, and monolayers were irradiated with unfiltered 14 W fluorescent light 6 cm from the surface of the well, which provided an incident fluence rate of 0.2 mW $cm^{-2}$. The MEM without phenol red was replaced with α-MEM plus 10% FBS, and cells were incubated for 24 h at 37°C in the dark. Cells were then trypsinised and counted using a particle counter (Coulter ZM, Coulter Electronics, Hialeah, FL, USA). Data are expressed as the cell number obtained from cultures irradiated in the presence of PPS expressed as a percentage of control (cells plus PPS not irradiated or irradiated cells minus PPS).

Uptake of [$^{14}$C]PII into cultured tumour cells was estimated using cultures parallel to those above by adding various concentrations of [$^{14}$C]PII and incubating cells in the dark for 24 h. The medium containing labelled material was removed, cells were trypsinised with 0.2 ml of trypsin and radioactivity counted 24 h after transfer to 4 ml of Ecoscint, using the equipment described above.

### *Histological preparation and light microscopy*

The freshly excised tumours and their adjacent soft tissue were immediately placed on ice and examined. Tumour size and gross morphological features were recorded. Specimens were grossly cross-sectioned at 1–2 mm intervals and serially labelled. When enough tissue was available, representative sections were snap frozen at −70°C for future use. Material for light microscopic analysis was fixed in 10% neutral-buffered formalin for 20–24 h, processed on a Tissue-Tek automated processor, paraffin embedded, and sectioned at 6 μm intervals. Multiple serial sections throughout the tumour mass were stained with haematoxylin and eosin or Masson trichrome and examined by light microscopy.

### Results

### *Effect of PDT on growth of mesotheliomas or R3230AC tumours in the nude mouse*

The response of mesothelioma or R3230AC xenografts to PDT was studied using a variety of irradiation protocols delivered 24 h after administration of 5 mg $kg^{-1}$ PPS. All PDT light treatments applied to either neoplasm produced significant delays ($P < 0.004$) in tumour volume doubling time compared with untreated controls (no treatment, group 1; Tables I and II).

Analyses of the tumour growth data for treatment of mesotheliomas by PDT (Figure 1a and Tables I and III) demonstrated fluence dependence when light was delivered at a fluence rate of 50 mW $cm^{-2}$; there was a significant increase in tumour volume doubling time when 90 J $cm^{-2}$ was compared with 180 J $cm^{-2}$, and a nearly significant increase ($P = 0.053$) from 180 J $cm^{-2}$ to 270 J $cm^{-2}$ total fluence, while no difference was observed when 270 was compared with 360 J $cm^{-2}$. All light treatments delivered continuously at this dose rate, i.e. 50 mW $cm^{-2}$, except for animals that received a total fluence of 90 J $cm^{-2}$, produced a delay in mesothelioma growth that was significantly greater than that seen in animals receiving a total fluence of 360 J $cm^{-2}$ delivered at a rate of 200 mW $cm^{-2}$. Similar relationships between extent of tumour growth delay and fluence rate or total fluence were not as evident for the R3230AC xenografts exposed to PDT (Figure 1b and Tables II and III). Although all light treatment protocols provided significant delays in tumour growth when compared with controls, no fluence rate or total fluence dependence was detected for the R3230AC tumour when intercomparisons of irradiation protocols were analysed. Comparisons of the effects of PDT on mesothelioma *vs* R3230AC xenografts, however, showed significantly greater control of the mesothelioma for all the total fluences except 180 J $cm^{-2}$ when light was delivered at 50 mW $cm^{-2}$ or when 360 J $cm^{-2}$ was applied using the fractionated protocol with a fluence

**Table I** Response of mesothelioma tumours to PDT

| Group | *Irradiation* Fluence rate ($mW\,cm^{-2}$) | Fluence ($J\,cm^{-2}$) | *Response* Days to double initial volume (number of mice) | Number of tumours doubling out of total | Median[a] (days) |
|---|---|---|---|---|---|
| 1 | 0 | 0 | 1(4), 2(3), 3(7), 4(3), 5(1), 7(3) | 21/21 | 3 |
| 2 | 200 | 360 | 9(2), 13(1), 17(1), 21(1), 23(1), 25(1), 28[b](2) | 7/9 | 21 |
| 3 | 50 | 90 | 5(1), 11(1), 12(1), 15(1), 17(2), 25(1) | 7/7 | 15 |
| 4 | 50 | 180 | 21(1), 30(1), 31(1), 44(1), 55(1), 58[b](1), 70[b](1) | 5/7 | 44 |
| 5 | 50 | 270 | 44[b](2), 46[b](1), 64[b](1), 84[b](1) | 0/5 | >46 |
| 6 | 50 | 360 | 26[b](1), 28(1), 33[b](1), 37[b](1) 38[b](1), 40[b](3), 44[b](1), 61[b](1), 69[b](1) | 1/11 | >40 |
| 7 | 100 | 360[c] | 49(1), 195(1), 208[b](1), 237[b](1), 278[b](1), 280[b](1), 371[b](1) | 2/7 | >237 |

[a]Median number of days for tumours to reach twice initial volume. Values are expressed as greater than for groups in which fewer than half of the tumours were observed to double in volume. [b]Number of post-treatment observation days for tumours that did not double in volume. [c]Irradiation fractionated at 30 s on/30 s off (see Materials and methods).

**Table II** Response of R3230AC tumour xenografts to PDT

| Group | *Irradiation* Fluence rate ($mW\,cm^{-2}$) | Fluence ($J\,cm^{-2}$) | *Response* Days to double initial volume (number of mice) | Number of tumours doubling out of total | Median[a] (days) |
|---|---|---|---|---|---|
| 1 | 0 | 0 | 2(4), 3(5), 4(4) | 13/13 | 3 |
| 2 | 200 | 360 | 13(1), 15(2), 17(1), 19(1), 29(1) | 6/6 | 16 |
| 3 | 50 | 90 | 4(1), 7(1), 8(2), 10(1), 12(1) | 6/6 | 8 |
| 4 | 50 | 180 | 7(1), 8(1), 12(1), 13(1), 14(1), 14[b](3) | 5/8 | 14 |
| 5 | 50 | 270 | 3(1), 5(2), 12(2), 17(1), 21(1) | 7/7 | 12 |
| 6 | 50 | 360 | 2(1), 3(1), 4(1), 5(1), 12(1), 15(1), 16(2), 17(1), 18(2), 44(1) | 12/12 | 15 |
| 7 | 100 | 360[c] | 11(1), 16(1), 18(1), 20(1), 31(1), 36(1), 38(1) | 7/7 | 20 |

[a]Median number of days for tumours to reach twice initial volume. [b]Number of post-treatment observation days for tumours that did not double in volume. [c]Irradiation fractionated at 30 s on/30 s off (see Materials and methods).

**Figure 1** Effects of PDT on the growth of mesothelioma (**a**) or R3230AC (**b**) tumours implanted in the flanks of nude mice. Data represent the percentage of tumours that reached twice the initial pretreatment volume. The groups in **a** (mesothelioma) and **b** (R3230AC; see Tables I and II) are control, no PPS, no light (no. 1, O), or treatment groups in which light was delivered at 200 $mW\,cm^{-2}$ for 30 min = 360 $J\,cm^{-2}$ (no. 2, □), at 50 $mW\,cm^{-2}$ for 30, 60, 90 or 120 min equalling 90 (no. 3, Δ), 180 (no. 4, ■), 270 (no. 5, ●) or 360 $J\,cm^{-2}$ (no. 6, ▲) or at 100 $mW\,cm^{-2}$ for 2 h = 360 $J\,cm^{-2}$ fractionated with 30 s on and 30 s off intervals (no. 7, X). All tumour-bearing animals in the treatment groups were injected i.p. with 5 $mg\,kg^{-1}$ PPS 24 h prior to exposure of lesions to visible laser light.

rate of 100 $mW\,cm^{-2}$. The observed difference in response of these two xenografts to the various PDT light regimens suggests that certain intrinsic properties of neoplasms may be important determinants of the efficacy of PDT.

### *Uptake and retention of [$^{14}$C]polyhaematoporphyrin in tumour and normal tissues*

One possible explanation for the enhanced tumour response of mesothelioma *vs* R3230AC xenografts could be a difference in the amount of porphyrin retained in these tumours. To address this, we examined [$^{14}$C]PII uptake and retention in tumours and in selected normal tissues from mice bearing either tumour type, so as to eliminate any bias attributable to the presence of one tumour or the other. The data (Figure 2) show that there were decreases in radioactivity over a 48 h period in liver, heart and lung tissue. The radioactivity in mesothelioma and R3230AC xenografts did not change significantly during the same time course. Skin, however, displayed an increase in radioactivity in samples taken at 24 or 48 h after [$^{14}$C]PII was injected when compared with the 4 h time point. Thus, both tumour xenografts accumulated and/or retained similar amounts of $^{14}$C-labelled porphyrin, whereas normal tissues displayed a greater diversity in the patterns of uptake and release of PII during the time course studied.

### In vivo *uptake of [$^{14}$C]polyhaematoporphyrin into tumour and liver mitochondria*

Although there was no difference in the total amount of radioactivity found in mesothelioma or R3230AC tumour homogenates, it is possible that the subcellular distribution of sensitiser could differ between these tumours and that this might offer an explanation for the differences in their response. To examine this possibility, we used differential centrifugation to determine the amount of radiolabelled porphyrin present in subcellular fractions of both xenografts and liver obtained 24 h after injection of [$^{14}$C]PII *in vivo*. The data summarised in Table IV demonstrate that there was no significant difference in the radioactive content of any of the subcellular fractions when the two tumour preparations were compared after undergoing the same differential centrifugation procedures. These data indicate that subcellular distribution of [$^{14}$C]PII components was equivalent for both tumours and should not account for the difference in response of these xenografts to PDT. The protein content of each subcellular fraction from the two xenografts was almost identical. It is interesting to note that the data in Table IV demonstrate that the accumulation of radiolabelled porphyrin in liver mitochondria was about 2–3 times that found in the whole-liver homogenate indicating that liver mitochondria concent-

**Table III** Statistical analysis of tumour response to PDT

| Mesothelioma (I) Groups | P-values | R3230AC (II) Groups | P-values | Mesotheliom (I) vs R3230AC (II) Groups | P-values |
|---|---|---|---|---|---|
| [a]I-1 *vs* I-2 to I-7 All | **0.0001**[b] | II-1 *vs* II-2 to II-7 | **≤0.0042** | I-1 *vs* II-1 | 0.4435 |
| I-2 *vs* I-3 | 0.1795 | II-2 *vs* II-3 | **0.0022** | I-2 *vs* II-2 | 0.6102 |
| I-4 | **0.0411** | II-4 | 0.1792 | I-3 *vs* II-3 | **0.0256** |
| I-5 | **0.0210** | II-5 | 0.1760 | I-4 *vs* II-4 | 0.0740 |
| I-6 | **0.0011** | II-6 | 0.5442 | I-5 *vs* II-5 | **0.0025** |
| I-7 | **0.0040** | II-7 | 0.1457 | I-6 *vs* II-6 | **<0.0001** |
| | | | | I-7 *vs* II-7 | **0.0003** |
| I-3 *vs* I-4 | **0.0012** | II-3 *vs* II-4 | **0.0286** | | |
| I-5 | **0.0025** | II-5 | 0.3019 | | |
| I-6 | **0.0001** | II-6 | 0.1013 | | |
| I-7 | **0.0006** | II-7 | **0.0023** | | |
| I-4 *vs* I-5 | 0.0530 | II-4 *vs* II-5 | 0.4283 | | |
| I-6 | **0.0450** | II-6 | 0.7507 | | |
| I-7 | **0.0210** | II-7 | 0.1803 | | |
| I-5 *vs* I-6 | 0.9176 | II-5 *vs* II-6 | 0.6674 | | |
| I-7 | 1.0000 | II-7 | **0.0344** | | |
| I-6 *vs* I-7 | 0.7190 | II-6 *vs* II-7 | 0.1706 | | |

Values appearing in bold type designate those comparisons that were significant, $P < 0.05$. [a]I, mesothelioma; II, R3230AC, arabic numbers 1–7 are light treatment protocol groups from Tables I and II. [b]Significance determined using the log-rank test (*P*-values) as described in Materials and methods.

**Figure 2** Uptake of $^{14}$C-labelled PII into various tissues at 4, 24 or 48 h after i.p. administration (see Materials and methods). Radioactivity is presented as d.p.m. per 100 mg of tissue, mean ± s.e.m.

**Table IV** [$^{14}$C]PII content of subcellular fractions

| Sample | Mesothelioma | R3230AC | Liver |
|---|---|---|---|
| Homogenate | 377 (56) | 404 (93) | 2837 (33) |
| 800 *g* SN | 635 (88) | 497 (20) | 2751 (424) |
| 800 *g* Pellet | 205 (32) | 279 (78) | 1767 (201) |
| 8000 *g* SN | 398 (52) | 316 (67) | 1009 (106) |
| 15000 *g* SN | 426 (72) | 524 (100) | 870 (90) |
| Mitochondria | 598 (86) | 553 (67) | 6468 (529) |

Mice bearing either mesothelioma or R3230AC tumours were administered 6.25 mg kg$^{-1}$ $^{14}$C-labelled PII 24 h prior to sacrifice and removal of tumour and liver tissue. Subcellular fractions were prepared and radioactive content determined as described in Materials and methods. Data are expressed as d.p.m. × $10^{-1}$ per mg of protein as determined for each fraction. Values are the means of at least four separate determinations and the numbers in parentheses are the s.e.m. SN, supernatant.

rate [$^{14}$C]PII. Taken together, these data do not implicate differences in subcellular localisation of [$^{14}$C]PII as the basis for the greater sensitivity of mesothelioma xenografts to PDT.

### *Effects of PDT on mitochondrial cytochrome* c *oxidase* in vivo *and* in vitro

The effect of photosensitisation on the activity of cytochrome *c* oxidase was measured in tumour mitochondria (obtained and isolated 24 h after administration of PPS, 5 mg kg$^{-1}$) by exposing mitochondrial suspensions to visible irradiation *in vitro*. We previously demonstrated that cytochrome *c* oxidase activity in mesothelioma tumour homogenates was photosensitised to the greatest extent at 24–48 h after PPS injection (Feins *et al.*, 1990). Therefore, we selected 24 h as the time to compare efficacy of *in vitro* light exposure of mitochondria prepared from either mesothelioma or R3230AC xenografts. Inhibition of cytochrome *c* oxidase activity (Figure 3) in mitochondrial preparations from mesothelioma tissue was significantly greater than in similar preparations from R3230AC tumours. The initial rate of enzyme inhibition in irradiated mitochondria suspensions from mesotheliomas was 0.12% J$^{-1}$ cm$^{-2}$, whereas enzyme activity in R3230AC preparations was reduced by 0.08% J$^{-1}$ cm$^{-2}$. No inhibition of enzyme activity was observed in either tumour mitochondrial preparations containing no PPS and exposed to light or in suspensions containing PPS that were maintained in the dark. These data suggest a biochemical basis for the observed difference in response of these xenograft to PDT.

### *Effects of PPS photosensitisation on tumour mitochondria cytochrome* c *oxidase* in vitro

We exposed tumour mitochondria, prepared from untreated animals, to PPS (2.5 µg ml$^{-1}$) and light *in vitro*, in an effort to determine whether any intrinsic differences in photosensitivity of cytochrome *c* oxidase between these two neoplasms would be evident. The data we obtained (Figure 4) demonstrate no apparent difference in the photosensitivity of this mitochondrial enzyme for these two tumours.

### *Uptake of [$^{14}$C]polyhaematoporphyrin in cultured tumour cells*

Cultured mesothelioma or R3230AC cells were incubated with radioactively labelled [$^{14}$C]PII, and cellular uptake of the

$^{14}C$-labelled material was assessed after 1, 24 and 48 h of exposure. The amount of porphyrin accumulated in either cell line by 24 h after the addition of [$^{14}C$]PII was equivalent over a range of 0.5 to 10.0 $\mu g\ ml^{-1}$ of photosensitiser (Figure 5). Results obtained after 1 h or after 48 h incubation of these tumour cells *in vitro* (data not shown) demonstrated the same pattern of [$^{14}C$]PII uptake as seen after 24 h *in vitro*.

*Effects of PPS photosensitisation on the viability of cultured tumour cells*

In other experiments, cultured mesothelioma or R3230AC cells were exposed to various concentrations of PPS and

**Figure 3** Effects of irradiation on the activity of mitochondrial cytochrome *c* oxidase measured in suspensions prepared from tumours 24 h after *in vivo* administration of 5 mg $kg^{-1}$ PPS. Details of experimental procedures appear in Materials and methods. Data are presented as the percentage of initial enzyme activity (prior to irradiation) and each point represents the mean ± s.e.m. of at least eight separate experiments performed in duplicate for mesothelioma (O) or R3230AC (●). Initial cytochrome *c* oxidase activity was 0.4–0.6 μmol cytochrome *c* oxidised $min^{-1}$ $mg^{-1}$ mitochondrial protein.

**Figure 4** Effects of PPS-induced photosensitisation *in vitro* on mitochondrial cytochrome *c* oxidase measured in mesothelioma (O) or R3230AC (●) mitochondrial suspensions prepared from tumours implanted in nude mice (see Materials and methods). Data are presented as the percentage of initial enzyme activity. Each point represents the mean ± s.e.m. of at least four separate experiments.

either 2.5 or 5.0 min of fluorescent light (see Materials and methods). Viability was determined at 24 h after light exposure (Figure 6). The data indicate that the R3230AC cells were significantly more sensitive ($P<0.05$) to the higher doses of PPS (5 or 10 $\mu g\ ml^{-1}$) than the cultured mesothelioma cells. These data also show that, despite equivalent uptake of [$^{14}C$]PII by each cell line, photosensitised cytotoxicity was more pronounced in the R3230AC cell line *in vitro*. These observations contrast with the significantly greater efficacy of PDT on mesotheliomas *in vivo*.

*Histology of untreated mesothelioma and R3230AC xenografts*

To determine whether the morphology of these tumours differ, histological preparations were examined. Sections of the R3230AC mammary carcinoma show a subcutaneous tumour nodule that is well delineated from the surrounding loose connective tissue. As seen in a representative section

**Figure 5** Total accumulation of [$^{14}C$]PII of mesothelioma (O) or R3230AC (●) cultured cells after a 24 h incubation. Each data point represents the mean ± s.e.m. (ng of PII per μg of total cell protein) determined from four separate experiments performed in duplicate.

**Figure 6** Effects of PPS photosensitisation on cell viability of either mesothelioma or R3230AC cultured cells. Cells were exposed to 1, 5 or 10 $\mu g\ ml^{-1}$ PPS for 24 h prior to irradiation for 2.5 or 5.0 min (see Materials and methods). Data are presented as the percentage of control cell number (not exposed to PII or light, 0.85–1.0 × $10^6$ cells per well). Each data point represents the mean ± s.e.m. obtained from at least four separate experiments performed in duplicate.

depicted in Figure 7, this well- to moderately differentiated neoplasm is composed of variably sized glandular formations, as well as small nests of tumour cells. A thin fibrovascular stromal component, containing a network of capillaries and small arteriovenous channels, surrounds these tumour elements. The neoplastic cells range from columnar to cuboidal forms, which have eosinophillic vesicular cytoplasm, coarse nuclear chromatin, and one to occasionally multiple nucleoli. An average of seven mitotic figures is present per 40 × microscopic field. Tumour vessel lumen dimensions are highly variable, with arteriovenous branches as wide as 60–80 μm in diameter present throughout the tumour volume.

The H-MESO-1 tumour consists of trabeculae and papillary tumour fronds, which are generally found surrounding a fibrovascular core. This structure is well depicted in the section presented in Figure 8. The radius of the viable tumour cell chords is typically around 140 μm. Beyond the viable cells, necrotic and degenerating tissue is found using either haematoxylin and eosin or Masson trichrome stain. Capillaries supplying the fronds appear quite uniform in diameter (10–15 μm), and larger vessels are rare and confined to the tumour periphery. With the Masson trichrome stain, contrast is excellent between the darkly stained red cells within the vessel lumen and the surrounding tumour cells. In some larger tumours, an irregular region of cavitating necrosis is present near the centre of the lesion. Focal small clusters of lymphoid inflammatory infiltrate are found predominantly at the tumour periphery. The neoplastic cells contain a moderate amount of eosinophilic cytoplasm, moderately pleomorphic nuclei and a spectrum of indistinct to multiple prominent nucleoli. Individual tumour cell necrosis and detritus can be found. The average mitotic rate is approximately 13 per 40 × microscopic field.

**Figure 7** Histological section of an untreated subcutaneous R3230AC mammary carcinoma excised from a nude mouse. Tissue was stained with haematoxylin and eosin and photographed under bright-field illumination (field size = 350 μm × 230 μm). The clear, unstained regions are variable-sized glandular formations, and a perfused vessel is present in the centre of the field.

**Figure 8** Histological section of an untreated human mesothelioma xenograft excised from a nude mouse. The tissue was stained with Masson trichrome and photographed under bright-field illumination through a cyan filter (Kodak Wratten, gelatin no. 44), with the field size identical to that of Figure 7a. The darkly stained perfused vessel in the centre of the photographic field supplies a well-defined chord of viable tumour cells.

### Discussion

Patients with diffuse malignant mesothelioma (DMM), a disease that invades the pleural and peritoneal cavities, have an expected survival of less than 2 years (Law *et al.*, 1984; Alberts *et al.*, 1988; Kraup-Hansen & Hansen, 1991). Failure to achieve favourable response to current treatments may be due to the inability to detect and treat early disease stages. The potential offered by PDT, and the fact that DMM presents as small tumour nodules embedded in a sheet of malignant cells, suggests that DMM may respond to PDT. In a previous report (Feins *et al.*, 1990), we found that PDT inhibited growth of human mesothelioma xenografts, with response inversely related to fluence rate, i.e. 360 J $cm^{-2}$ delivered at 50 mW $cm^{-2}$ was more effective than the same fluence administered at fluence rates of 100 or 200 mW $cm^{-2}$. Under the conditions employed in this earlier study using 50 mW $cm^{-2}$, a total fluence of 180 J $cm^{-2}$ appeared to be as effective as 270 or 360 J $cm^{-2}$ in controlling tumour growth. We found similar results when we extended the observation time in the present study.

Although we observed a dramatic reduction in tumour growth rate, which, in comparison with data obtained for the R3230AC mammary tumours in the rats (Gibson *et al.*, 1990), showed that the mesothelioma was more sensitive, a number of questions remained and formed the basis for our current studies. These questions were: (a) would the suppression of mesothelioma tumour growth be maintained over a longer period of time, since our previous report consisted of an 18 day post-PDT observation time (Feins *et al.*, 1990); (b) was this mesothelioma peculiarly sensitive to PDT because it was studied as a xenograft?; (c) was the better response of mesothelioma to PDT intrinsic to this tumour model?

From the data (Tables I and III, Figure 1a), it is apparent that prolonged mesothelioma tumour response was achieved when irradiation at 50 mW $cm^{-2}$ fluence rate was delivered for total fluences of $>$180 J $cm^{-2}$. Under these conditions, tumour growth, i.e. tumour volume doubling, occurred at a median of 40 days or longer after PDT (see footnote to Table II), thus extending the previous observation period by 2–4 times. When the fractionated light treatment regimen that produced the best R3230AC tumour response in rats (Foster *et al.*, 1991) was applied to mesothelioma xenografts, striking responses were observed, with 5/7 mice showing no tumour volume doubling for observation periods of 208–371 days after PDT (Table I).

Although the fluence rate dependence of response to PDT for mesothelioma xenografts was similar to those for R3230AC mammary tumours in rats (Gibson *et al.*, 1990; Foster *et al.*, 1991), the tumour volume doubling time of mesothelioma was extended compared with the mammary tumour in the rat. These results raise the question of whether the observed difference in response was attributable to some intrinsic feature of the tumour or the host. To address the former, we also examined R3230AC tumours grown as xenografts in mice. These R3230AC xenografts did not respond as well as mesotheliomas and, surprisingly, their response was neither fluence nor fluence rate dependent as it was when they were treated in isologous hosts (Gibson *et al.*, 1990). In an effort to elucidate the mechanisms responsible for the different response of the two xenografts, we examined the time course of [$^{14}$C]PII uptake into both tumours; no difference was observed (Figure 2). However, when mitochondria from these xenografts were isolated and irradiated, cytochrome *c* oxidase from mesotheliomas was significantly more photosensitive (Figure 3). One explanation for this might be that the 'active' components of PPS localised in different subcellular sites in these two xenografts. This, however, was ruled out by the measurement of [$^{14}$C]PII distribution among the subcellular fractions (Table IV). This was further supported by studies of cultured tumour cells *in vitro*, in which the uptake of [$^{14}$C]PII was essentially the same for mesothelioma cells and R3230AC (Figure 5). Unexpectedly, the cytotoxicity of PPS-induced photosensitisation of cells in culture was not equal for both cell types at various sensitiser and light doses; in most instances, the R3230AC cells were significantly more sensitive. Thus, we find that differences in response to PDT *in vivo* and photosensitisation *in vitro* are not simply related to a difference in photosensitiser concentration or its mitochondrial localisation.

Ris *et al.* (1993) arrived at a conclusion coincidental with ours when they found that the tissue concentration of mTHPP did not correlate with mesothelioma xenograft response to PDT. These findings, combined, indicate that the level of photosensitiser sequestered in malignant tissue may not ultimately govern tumour response to PDT when studying either one tumour and different doses of photosensitisers or comparing two tumour types with the same concentration of intratumoral photosensitisers.

Others have studied various tumours and their response to PDT but, to our knowledge, none has compared two morphologically different tumours in the same host or the same tumour in different hosts. Henderson *et al.* (1985) reported that 90–100% of EMT6 tumours in Balb/c mice did not recur after PDT, whereas only 13% of RIF tumours in C3H/HeJ mice did not recur. They attributed this disparity in response to a difference in the degree of vascular damage, although they did not rule out occurrence of direct cell damage in EMT6 tumours. Nahabedian *et al.* (1988), confirming those findings, combined PDT with either cisplatin or adriamycin chemotherapy; adriamycin potentiated the PDT-induced response of EMT6 tumours but was only additive in RIF tumours. Here, we observed differences in the response to PDT of two xenografts of different origin (the R3230AC rodent mammary adenocarcinoma and a human H-MESO-1 mesothelioma) in the same host.

One explanation for the disparity in response of these two tumours to PDT might be that the host haemopoietic response differs. Canti *et al.* (1984) demonstrated increased marrow cellularity and splenic hyperplasia after various multiple daily injections of 8–75 mg kg$^{-1}$ haematoporphyrin derivative (HpD) without exposure of the host to light. Similar haemopoietic responses were observed by Henderson and Stewart (1992) and Levy *et al.* (1992) using Photofrin II. Those studies imply that there may be haemopoietic stimulation resulting from the porphyrin photosensitiser either *in vitro* or *in vivo* and that this stimulation may occur both in the dark and after exposure to light. In this present study, we utilised the nude mouse to xenograft both tumour types, and we would expect little difference in haemopoietic response because of a number of factors. Growth of both tumours was not significantly different from growth of xenografts in the nude mouse or from the growth of R3230AC in its isologous host, the Fischer female rats. Administration of 5 mg kg$^{-1}$ PPS was expected to elicit the same response, particularly since photosensitiser retention and distribution in the xenografted tumours were equivalent.

The results of the present study strongly suggest that PDT response in these systems cannot be predicted on the basis of easily identified 'cell-specific' or 'host-specific' criteria. Rather, it appears that complex factors involving tumour cell–host interactions are important and difficult to analyse. R3230AC cells, while more responsive to PDT than H-MESO-1 cells *in vitro*, are clearly less responsive to equivalent PDT treatment regimens in both isologous hosts and nude mice *in vivo*. Hence, sensitivity to PDT *in vitro* is not an invariable predictor of the tumour sensitivity *in vivo*. Further, these two tumour cell lines, when grown as subcutaneous transplants in the same host, exhibit significantly different responses to PDT. Thus, we have been unable to identify any obvious feature of the host as being responsible for tumour response.

The complexity of this is further illustrated by the fact that the R3230AC tumour exhibits a different fluence and fluence rate dependence in the two host systems. In the Fischer rat, significant prolongation of tumour regrowth is observed when reduced fluence rate or fractionated irradiation protocols are employed, indicating that therapy-induced oxygen consumption is an important limiting factor in this system. On the other hand, no such fluence rate dependence is observed for this tumour when treated as a xenograft in the nude mouse. It is possible that the R3230AC elicits a better vascular supply when grown in the mouse and that this better perfusion is responsible for the absence of a fluence rate effect (Foster & Gao, 1992). However, it is also possible that some other factor plays the predominant limiting role. If, for example, the intratumoral distribution of PPS is significantly more heterogeneous in the xenograft system than it is in the isologous host, it may not be possible to influence the tumour response to PDT by reduction in the rate of photochemical oxygen consumption. Differences in intratumoral photosensitiser distribution would not be measured by the $^{14}$C uptake studies and biochemical assays reported here, since these represent spatial averages over the entire tumour volume. Whatever the mechanism governing the lack of a fluence rate dependence for response of the R3230AC in the mouse, it is clear that it cannot be attributable to an intrinsic property of the murine host, since pronounced fluence rate effects are observed in the mesothelioma grown in this host system.

Examination of histological sections of the two xenografts demonstrates that their morphologies are very different. Generally, the mesothelioma appears to consist of a uniform distribution of cells and vessels. The R3230AC, on the other hand, demonstrates little uniformity in either cellular distribution or vessel location and diameter. Whether these structural variations are the basis for the significant differences in response of these tumours to PDT is, as yet, unknown.

In conclusion, we find that the sensitivity of a human mesothelioma xenograft to PDT is dependent on total fluence and inversely dependent on incident fluence rate. These mesothelioma xenografts are significantly more responsive to PDT at a lower fluence rate and high total fluences than are rodent R3230AC tumour xenografts in the same host. The basis for the observed difference in sensitivity of these two xenografts could not be attributed directly to photosensitiser uptake or its subcellular localisation or to any intrinsic sensitivity studied here.

This study was supported by USPHS Grant CA38656, the National Institutes of Health and the American Lung Association of New York State. We also acknowledge the Animal Tumor Research Facility of the University of Rochester Cancer Center (CA11198) for transplantation and maintenance of the H-MESO-1 and R3230AC tumour lines.

### References

ALBERTS, A.S., FALKSON, G., GOEDHALS, L., VOROBIOF, D.A. & VAN DER MERWE, C.A. (1988). Malignant pleural mesothelioma: a disease unaffected by current therapeutic maneuvers. *J. Clin. Oncol.*, **6**, 527–535.

BERG, K. & MOAN, J. (1988). Photodynamic effects of Photofrin II on cell division in human NHIK 3025 cells. *Int. J. Radiat. Biol.*, **53**, 797–811.

CANTI, G., FRANCO, P., MARELLI, O., RICCI, L. & NICOLIN, A. (1984). Hematoporphyrin derivative rescue from toxicity caused by chemotherapy or radiation in a murine leukemia model (L1210). *Cancer Res.*, **44**, 1551–1556.

DOUGHERTY, T.J. & MANG, T.S. (1987). Characterization of intratumoral porphyrin following injection of hematoporphyrin derivative or its purified component. *Photochem. Photobiol.*, **46**, 67–70.

FEINS, R.H., HILF, R., ROSS, H. & GIBSON, S.L. (1990). Photodynamic therapy for human malignant mesothelioma in the nude mouse. *J. Surg. Res.*, **49**, 311–314.

FOSTER, T.H. & GAO, L. (1992). Dosimetry in photodynamic therapy: oxygen and the critical importance of capillary density. *Radiat. Res.*, **130**, 379–383.

FOSTER, T.H., MURANT, R.S., BRYANT, R.G., KNOX, R.S., GIBSON, S.L. & HILF, R. (1991). Oxygen consumption and diffusion effects in photodynamic therapy. *Radiat. Res.*, **126**, 296–303.

GIBSON, S.L. & HILF, R. (1983). Photosensitization of mitochondrial cytochrome *c* oxidase by hematoporphyrin derivative and related porphyrins *in vitro* and *in vivo*. *Cancer Res.*, **43**, 4191–4197.

GIBSON, S.L., COHEN, H.J. & HILF, R. (1984). Evidence against the production of superoxide by photoirradiation of hematoporphyrin derivative. *Photochem. Photobiol.*, **40**, 441–448.

GIBSON, S.L., VAN DER MEID, K.R., MURANT, R.S., RAUBERTAS, R.F. & HILF, R. (1990). Effects of various photoradiation regimens on the antitumor efficacy of photodynamic therapy for R3230AC mammary carcinomas. *Cancer Res.*, **50**, 7236–7241.

GOMER, C.J. & DOUGHERTY, T.J. (1979). Determination of [$^{3}$H]- and [$^{14}$C]-hematoporphyrin derivative distribution in malignant and normal tissue. *Cancer Res.*, **39**, 146–151.

HENDERSON, B.W. & STEWART, C.C. (1992). Effects of porphyrins on murine hematopoiesis. *Photochem. Photobiol.*, **555**, 75.

HENDERSON, B.W., WALDOW, S.M., MANG, T.S., POTTER, W.R., MALONE, P.B. & DOUGHERTY, T.J. (1985). Tumor destruction and kinetics of tumor cell death in two experimental mouse tumors following photodynamic therapy. *Cancer Res.*, **45**, 572–576.

HILF, R., MURANT, R.S., NARAYANAN, U. & GIBSON, S.L. (1986). Relationship of mitochondrial function and cellular adenosine triphosphate levels to hematoporphyrin derivative-induced photosensitization in R3230AC mammary tumors. *Cancer Res.*, **46**, 211–217.

HILF, R., GIBSON, S.L., PENNEY, D.P., CECKLER, T.L. & BRYANT, R.G. (1987). Early biochemical responses to photodynamic therapy monitored by NMR spectroscopy. *Photochem. Photobiol.*, **46**, 809–817.

HISSIN, P.J. & HILF, R. (1978). Effect of insulin *in vivo* and *in vitro* on amino acid transport into cells from R3230AC mammary adenocarcinoma and their relationship to tumor growth. *Cancer Res.*, **38**, 3646–3651.

KEENE, J.P., KESSEL, D., LAND, E.J., REDMOND, R.W. & TRUSCOTT, T.G. (1986). Direct detection of singlet oxygen sensitized by hematoporphyrin and related compounds. *Photochem. Photobiol.*, **43**, 117–121.

KELLER, S.M., TAYLOR, D.D. & WEISE, J.L. (1990). *In vitro* killing of human malignant mesothelioma by photodynamic therapy. *J. Surg. Res.*, **48**, 337–340.

KESSEL, D. & CHENG, M.-L. (1985). On the preparation and properties of dihematoporphyrin ether, the tumor-localizing component of HPD. *Photochem. Photobiol.*, **41**, 277–282.

KRAUP-HANSEN, A. & HANSEN, H.H. (1991). Chemotherapy in malignant mesothelioma: a review. *Cancer Chemother. Pharmacol.*, **28**, 319–330.

LAW, M.R., GREGOR, A., HODSON, M.E., BLOOM, H.J.G. & TURNER, WARWICK, M. (1984). Malignant mesothelioma of the pleura: a study of 52 treated and 64 untreated patients. *Thorax*, **39**, 255–259.

LEVY, J.G., HUNT, D.W.C., MITCHELL, D.W. & JAMIESON, H.M. (1992). Hematopoietic stimulation by porphyrin photosensitizers. In *Optical Methods for Tumor Treatment and Detection*, Dougherty, T.J. (ed.), pp. 67–71. SPIE: International Society for Optical Engineering, Vol. 1645: Bellingham, WA.

LOWRY, O.H., ROSEBROUGH, N.J., FARR, A.L. & RANDALL, R.J. (1951). Protein measurement with the Folin phenol reagent. *J. Biol. Chem.*, **193**, 265–275.

MCDONALD, A.D. & MCDONALD, J.C. (1980). Malignant mesothelioma in North America. *Cancer*, **46**, 1650–1656.

MOAN, J., CHRISTENSEN, T. & SOMMER, S. (1982). The main photosensitizing components of hematoporphyrin derivative. *Cancer Lett.*, **15**, 161–168.

MUSCAT, J.E. & WYNDER, E.L. (1991). Cigarette smoking, asbestos exposure and malignant mesothelioma. *Cancer Res.*, **51**, 2263–2267.

NAHABIDIAN, M.Y., COHEN, R.A., CONTINO, M.F., FERENS, T.M., WRIGHT, W.H., BERNS, M.W. & WILE, A.G. (1988). Combination cytotoxic chemotherapy with cisplatin or doxorubicin and photodynamic therapy in murine tumors. *J. Natl Cancer Inst.*, **80**, 739–744.

PASS, H.I., TOCHNER, Z., DELANEY, T., SMITH, P., FRIAOF, W., GLATSTEIN, E. & TRAVIS, W. (1990). Intraoperative photodynamic therapy for malignant mesothelioma. *Ann. Thorac. Surg.*, **50**, 684–-S.

PERRY, R.R., MATTHEWS, W., MITCHELL, J.B., RUSSO, A., EVANS, S. & PASS, H.I. (1990). Sensitivity of different human lung cancer histologies to photodynamic therapy. *Cancer Res.*, **50**, 4272–4276.

RIS, H.-B., ALTERMATT, H.J., UNDERBITZI, R., HESS, R., NACHBUS, B., STEWART, J.C.M., WANG, Q., LIM, C.K., BONNETT, R., BERENBAUM, M.C. & ALTHAUS, U. (1991). Photodynamic therapy with chlorins for diffuse malignant mesothelioma: initial clinical results. *Br. J. Cancer*, **64**, 1116–1120.

RIS, H.-B., ALTERMATT, H.J., NACHLAUS, B., STEWART, J.C.M., LIM, C.K., BONNETT, R. & ALTHAUS, U. (1993). Effect of drug light interval on photodynamic therapy with meta-tetrahydroxyphenylchlorin in malignant mesothelioma. *Int. J. Cancer*, **53**, 141–146.

WAGNER, J.C., SLEGGS, C.A. & MARCHAND, P. (1960). Diffuse pleural mesothelioma and asbestos exposure in the North Western Cape Province. *Br. J. Indust. Med.*, **17**, 260–271.

WEISHAUPT, K.R., GOMER, C.J. & DOUGHERTY, T.J. (1976). Identification of singlet oxygen on the cytotoxic agent in photoinactivation of a murine tumor. *Cancer Res.*, **36**, 2326–2329.

WOODBURN, K.W., STYLLI, S., KAYE, A.H., REISS, J.A. & PHILLIPS, D.R. (1992). Evaluation of tumor and tissue distribution of porphyrin for use in photodynamic therapy. *Br. J. Cancer*, **65**, 321–328.